# Effects of water management and cultivar on carbon dynamics, plant productivity and biomass allocation in European rice systems

**DOI:** 10.1016/j.scitotenv.2019.06.110

**Published:** 2019-10-01

**Authors:** Viktoria Oliver, Nicole Cochrane, Julia Magnusson, Erika Brachi, Stefano Monaco, Andrea Volante, Brigitte Courtois, Giampiero Vale, Adam Price, Yit Arn Teh

**Affiliations:** a Institute of Biological Sciences, University of Aberdeen, Cruickshank Building, St. Machar Drive, AB24 3UU Aberdeen, UK; b Consiglio per la Ricerca in Agricoltura e l'analisi dell’ Economia Agraria (CREA), Centro di ricerca cerealicoltura e colture industriali, S.S.11 to Torino, 13100 Vercelli, Italy; c Department of Life Sciences and Systems Biology, University of Torino, Via Accademia Albertina, 13, Torino, Italy; d Centre de coopération internationale en recherche agronomique pour le développement (CIRAD), UMR AGAP, Avenue Agropolis, TA A-108/03, 34398 Montpellier, France

**Keywords:** Gross primary productivity, Net ecosystem exchange, Decomposition, Above and below ground biomass, Alternate wetting and drying, European rice cultivation

## Abstract

Water saving techniques, such as alternate wetting and drying (AWD), are becoming a necessity in modern rice farming because of climate change mitigation and growing water use scarcity. Reducing water can vastly reduce methane (CH_4_) emissions; however, this net climate benefit may be offset by enhanced carbon dioxide (CO_2_) emissions from soil. The main aims of this study were: to determine the effects of AWD on yield and ecosystem C dynamics, and to establish the underlying mechanistic basis for observed trends in net ecosystem C gain or loss in an Italian rice paddy. We investigated the effects of conventional water management (i.e. conventionally flooded paddy; CF) and AWD on biomass accumulation (aboveground, belowground, grain), key ecosystem C fluxes (net ecosystem exchange (NEE), net primary productivity (NPP), gross primary productivity (GPP), ecosystem respiration (ER), autotrophic respiration (RA), heterotrophic respiration (RH)), and soil organic matter (SOM) decay for four common commercial European rice cultivars. The most significant finding was that neither treatment nor cultivar affected NEE, GPP, ER or SOM decomposition. RA was the dominant contributor to ER for both CF and AWD treatments. Cultivar and treatment affected the total biomass of the rice plants; specifically, with greater root production in CF compared to AWD. Importantly, there was no effect of treatment on the overall yield for any cultivar. Possibly, the wetting-drying cycles may have been insufficient to allow substantial soil C metabolism or there was a lack of labile substrate in the soil. These results imply that AWD systems may not be at risk of enhancing soil C loss, making it a viable solution for climate change mitigation and water conservation. Although more studies are needed, the initial outlook for AWD in Europe is positive; with no net loss of soil C from SOM decomposition, whilst also maintaining yield.

## Highlights

• Alternate wetting and drying does not enhance carbon loss in European rice paddies.
• Aerobic conditions do not increase soil organic matter decomposition.
• Periods of drought do not affect rice yields in four European rice cultivars.
• Alternate wetting and drying reduces rice root production.

## Graphical abstract

**Figure:**
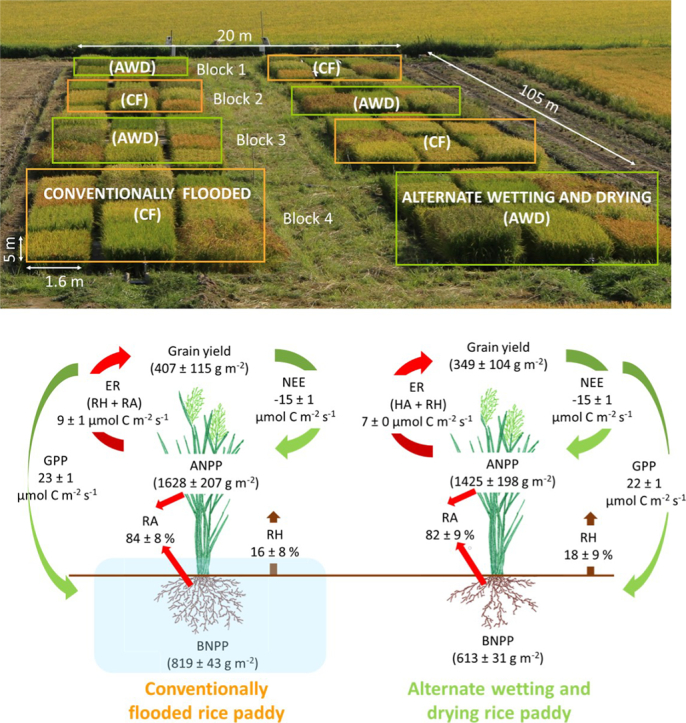

## 1 Introduction

Irrigated rice (*Oryza sativa* L.) is the largest consumer of water in the agricultural sector (Thakur et al., 2014) and can require up to 2500 L of water per kg yield, depending on the rice ecosystem and local climate (Bouman, 2009). In contrast, wheat and corn use on average 650–900 L per kg (Pimentel et al., 2004). Globally, 85–90 million ha of irrigated rice provides 75% of the world's rice production, supplying a major staple food for much of the world's population (IRRI, 2010; Seck et al., 2012). However, the rise in extreme heat and drought occurrence, combined with increasing populations, economic growth and diminishing water quality is intensifying the competition among agriculture, industry and urban populations for finite water supplies (Bates et al., 2008; Hanjra and Qureshi, 2010). For example, in Europe, irrigation is an essential element in many types of agricultural production, such as potatoes in northern Europe and cotton and maize in southern Europe (Baldock et al., 2000; “European Union (EU) agri-environmental indicator,”, 2019), and thus comprises a significant proportion of the total freshwater demand, with approximately 55% of consumptive water used in the agriculture sector (Bartram et al., 2002). Although the total European rice contribution is only 0.4% of the total global figure (FAO, 2014; USDA, 2015), it has economic, sociocultural and ecological importance in several Mediterranean countries, including the Ebro Delta in Spain, Rhone Delta in France and Lombardy in Italy. In these regions, not only does rice production contribute to local economies, but rice fields play a key role in managing local ornithological fauna populations and macroinvertebrate communities (Faure and Mazaud, 1995; Ibáñez and Caiola, 2018; Longoni, 2010; Lupi et al., 2013), and the harvested area is continually expanding (Ferrero, 2007; Ferrero and Vidotto, 2010). Thus, there is an urgent need to adopt strategies and practices that will use water efficiently for the future of irrigated rice production in Europe.

An equally concerning consequence of conventional flooded rice is the associated methane (CH_4_) emissions, which occur as a by-product of anaerobic decomposition of plant residues and soil organic matter (SOM). Subsequently, rice paddies account for 11% of the total global anthropogenic CH_4_ emissions (FAO, 2011; Smith et al., 2014), which is four times higher than for other major cereal crops, such as wheat or maize (Linquist et al., 2012). With global efforts to mitigate against climate change, reducing greenhouse gas emissions (GHG) from agricultural practices, such as rice production, is an integral part of the strategy to stabilize climate (IPPC, 2015). As a result, there have been considerable efforts to determine if aerobic cultivation or intermittent flooding are viable alternatives for maintaining high rice yields, yet simultaneously reducing CH_4_ emissions (Bouman and Tuong, 2001). One of the most recent and successful advances is a system of water managed called Alternate Wetting and Drying (AWD). This approach uses a system of periodic inundation over the rice production cycle to reduce overall water use and CH_4_ emissions, while simultaneously ensuring that the rice crop receives sufficient water input during critical periods of the production cycle, so as to prevent negative impacts on yield and grain quality (Price et al., 2013). Namely, during specific parts of the vegetative growth cycle (i.e. tillering and stem elongation), rice fields are allowed to drain naturally and are only re-wetted when the soil water level drops below 15 cm from the soil surface (designed to reflect a soil matric potential of around −15–20 kPa at 5–10 cm depth and below critical physiological thresholds. In “safe” AWD (Lampayan et al., 2015), the fields are fully inundated once more for the reproductive phases of plant growth (i.e. panicle initiation and flowering), in order to promote high levels of grain production and the formation of good quality grain (Price et al., 2013). Numerous studies conducted throughout Asia and parts of North America have demonstrated that AWD can reduce CH_4_ emissions by 35–90%, and improve overall water-use efficiency by 35–63% (Chidthaisong et al., 2018; Chu et al., 2015; Linquist Bruce et al., 2014; Rejesus et al., 2011; Setyanto et al., 2018; Tran et al., 2018; Yang et al., 2017).

In the majority of field trials, grain yields are generally maintained (Yao et al., 2012) or even increased (Jiang et al., 2017; Norton et al., 2017a; Norton et al., 2017b; Norton et al., 2018; Yang et al., 2017). A recent meta-analysis based on 56 studies found that safe AWD generally does not impact yield when practiced either during the vegetative stage or the reproductive phase (Carrijo et al., 2017). Soil physical and chemical properties were highlighted as being important in maintaining crop yields under AWD practices. Specifically, yield response of plants grown under AWD performed better in more acidic soils and soils with a higher organic content (Carrijo et al., 2017). Accordingly, AWD is being promoted more widely in parts of the Indian sub-continent and Southeast Asia, particularly in regions where water resources are already scarce (IRRI, 2010). This includes countries such as: Bangladesh, Indonesia, Lao PDR, Philippines, Myanmar, Vietnam and Japan (IRRI, 2010). Yet, despite the uncertainty posed by climate change and the general scarcity of water resources in rice-producing regions of Europe, we know little about whether AWD is a viable alternative for European rice farmers. Region-specific knowledge is crucial for assessing the practical viability of this new management approach, because prior research suggests that the success of AWD is contingent upon local plant cultivars thriving under AWD, with poorly-adapted cultivars potentially showing a negative response to reduced water inputs (Matsunami et al., 2012; Sandhu et al., 2017).

However, while research on the effects of water management (including AWD) on yield, grain quality and CH_4_ flux in rice is relatively well-developed, much less is known about the effects of different water management practices on ecosystem C dynamics, including processes such as net primary production (NPP), ecosystem respiration (ER), soil organic matter (SOM) decomposition, and biomass allocation (Linquist et al., 2018; Sass and Fisher, 1997; Wassmann et al., 2009). Given that AWD represents a shift to more oxidizing soil conditions, one potential impact of AWD is it may accelerate the decay of plant residues and SOM, particularly during the vegetative growth phase of rice. This could lead to enhanced loss of SOM as CO_2_, particularly during AWD cycles, which could partially offset any climate gains made by a net reduction in CH_4_ emissions.

For example, the few studies which have quantified net ecosystem exchange (NEE) of CO_2_ from intermittently flooded paddy fields in Japan, China and the Philippines showed significantly greater CO_2_ emissions than continuously flooded paddy soils, implying higher ER (Alberto et al., 2014; Liu et al., 2013; Miyata et al., 2000). Yet whether the higher ER was the result of increased autotrophic respiration (RA) or enhanced heterotrophic respiration (i.e. accelerated SOM and plant residue decay; abbreviated RH) is still uncertain. Published studies have relied on micrometeorological methods (i.e. eddy covariance) to quantify NEE from *single-cultivar* (rather than multiple cultivar) studies, and were further limited by the fact that the investigators' choice of sampling methodology did not partition ER into its component fluxes (i.e. RA and RH) (Baldocchi, 2003). In order to gain deeper insight into the factors that could be regulating ecosystem C loss, it is critical to partition the principal ecosystem C fluxes such as NEE and ER into their component fluxes, such as gross primary productivity (GPP), RA and RH (Bhattacharyya et al., 2013; Falge et al., 2002). This is because changes to any of these component fluxes can influence the balance of soil C storage and CO_2_ emissions to the atmosphere. Thus, it is important to determine how these component fluxes vary under different forms of water management (e.g. CF – continuously flooded versus AWD – alternate wetting and drying), for different cultivars, and in response to changes in other key environmental variables (e.g. air temperature, soil temperature, soil moisture content).

Moreover, shifts in soil moisture and other environmental conditions during the vegetative growth phase could promote changes in plant growth and allocation which could have wider consequences for SOM formation and storage (Jobbágy and Jackson, 2000). For example, reduction in soil moisture availability could promote increased plant allocation to roots, deepening of the root profile, or shifts in plant root to shoot ratios, in-line with plant allocation theory (Bloom et al., 1985; Jobbágy and Jackson, 2000). This could have knock-on effects for how and where plant residues are returned to the soil, with long-term effects for incorporation of plant residues into SOM, and the overall vertical distribution of SOM stocks throughout the profile (Jobbágy and Jackson, 2000). Thus, it is critical that we develop a clearer understanding of how water management strategies like AWD affect not only the net C balance of rice systems but also how plant allocation and soil C shift in response to water management.

To address these knowledge gaps, we conducted a process-based field experiment that compared the effects of conventional paddy management (hereafter, continuously flooded rice or CF) and AWD on the C dynamics of four commercial cultivars common throughout Southern Europe. Specifically, we investigated the effects of water management and rice cultivar on the principal C fluxes (i.e. NEE, ER), and their components (i.e. GPP, RA, RH). We also explored how water management and rice cultivar influenced plant biomass production, including allocation to belowground (root) production, leaves, shoots and grain. We predict that poorly-adapted rice cultivars will respond negatively to AWD, with a net reduction in total net primary productivity (NPP). Moreover, we hypothesised that for individual cultivars:

Net ecosystem exchange (NEE) is more positive (i.e. greater net C loss) under AWD compared to CF due to increased ER under more aerobic soil conditions.

Ecosystem respiration (ER) is greater under AWD compared to CF due to enhanced heterotrophic respiration (RH) and organic matter decay.

Total net primary productivity (NPP) and grain yield is similar in AWD compared to CF.

Belowground NPP (BNPP) will be greater in AWD compared to CF, while aboveground NPP (ANPP) will show the opposite trend, in-line with plant resource allocation theory.

## 2 Methods and materials

### 2.1 Study site and sampling design

Field experiments took place at CREA-Centro di ricerca cerealicoltura e colture industriali, Vercelli (45°19′21.96″N, 8°22′24.07″E), former CREA-RIS, in the western area of the Po River valley, Italy. These fields have been under rice cultivation for the last 30 years, with irrigation waters coming from a network of channels during the growing season (May–September) and fields left fallow during the winter months. Rice straw is not incorporated into the fields after harvest. The climate in the Po valley is temperate and sub-continental, characterised by a summer mean annual temperature of ~23 °C and average annual precipitation of 1300 mm. The soils are old alluvial soils, and are classified as anthraquic eutrudept, coarse-loamy, mixed, non-acid, mesic (sand = 49%; silt = 42%; clay = %), derived from Quaternary yellow sediment, with a C:N content of 10:1, bulk density 1.2 (g cm^−3^) and a pH of 6.4 (Table 1).

**Table 1:** Soil properties from the experimental plot (*n* = 10). including: soil C and N (%), Bulk density g cm^−3^), C and N stocks (Mg C ha^−1^) and the C:N ratio. Standard errors indicate standard 1 error of the mean.

Depth (cm)	C %	N	Bulk density (g cm^−3^)	C stocks	N stocks	C:N
				(Mg C ha^−1^)		
0–10	1.4 ± 0.1	0.1 ± 0.0	1.2 ± 0.1	8.2 ± 1.4	0.9 ± 0.1	10 ± 1
10–20	1.4 ± 0.0	0.1 ± 0.0	1.2 ± 0.1	8.2 ± 2.6	0.8 ± 0.2	10 ± 3
20–30	0.9 ± 0.0	0.1 ± 0.0	1.2 ± 0.1	5.3 ± 0.9	0.6 ± 0.1	10 ± 1
30–40	0.7 ± 0.0	0.1 ± 0.0	1.2 ± 0.1	4.2 ± 0.6	0.4 ± 0.1	10 ± 1
0–40				25.9 ± 5.5	2.6 ± 0.5	

**Fig. 1:**
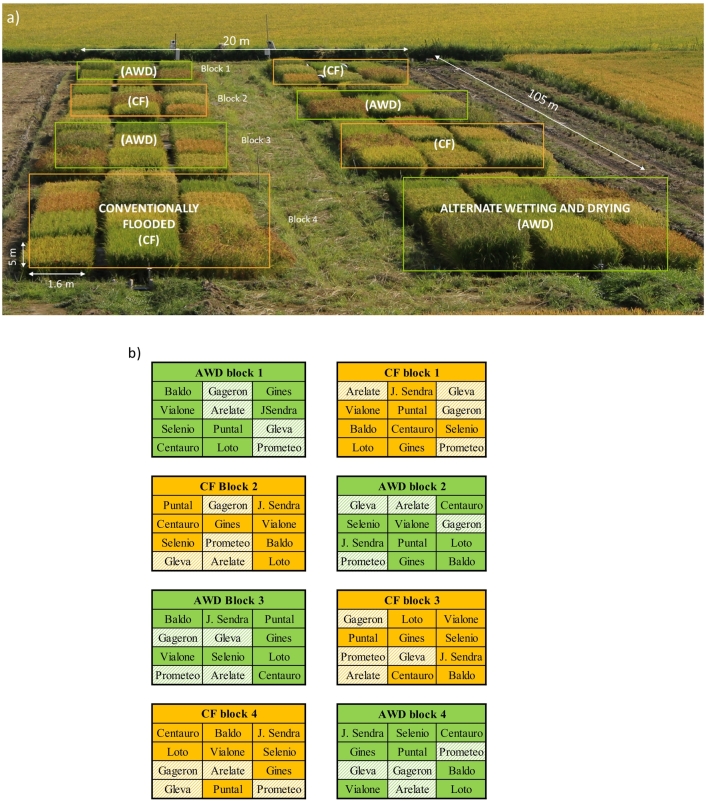
a. Top - split plot experimental design with four replicates (blocks) of continuously flooded (CF) in orange and alternate wetting and drying (AWD) plots in green. b. Bottom - each block and treatment randomly contained the four cultivars that were focused on in this study (Gleva, Arelate, Gageron and Prometeo) in shaded green (AWD) and orange (CF).

The experimental site (20 × 105 m), established in April 2017, was split into eight blocks, with an alternating paired design of four alternate wetting and drying (AWD) and continuously flooded (CF) replicates adjacent to each other (Fig. 1). Within each block, individual randomised plots of 12 European rice cultivars (1.6 × 5 m) (Fig. 1a and b). The rice cultivars included accessions from Italy (Baldo, Vialone nano, Selenio, Centauro, Loto, and Prometeo), France (Gageron, Gines, and Arelate) and Spain (J.Sendra, Puntal, and Gleva). In this study, the experimental sampling was conducted on: Arelate, Prometeo, Gleva and Gageron. Gleva and Prometeo are medium-grain rice, Arelate is long-grain rice and Gageron is short-grain rice. Agricultural and water management practices are summarized in Table 2. In brief, all plots were fertilised pre-sowing on the 26th April with a commercial dry manure (rate: 260 kg ha^−1^, total N content: 12.5%). Top-dress fertilization was then added on the 30th June (300 kg ha^−1^, 20–0-30). During the vegetative growth cycle (tillering, stem elongation), half the plots were subject to AWD with details of this water management provided in Section 2.2.

**Table 2:** Dates of the agricultural practices and water management that took place during the growing season. Variables include: the date when each agricultural practice and water management took place, the day since sowing, the product used and its commercial product rate, active ingredient and applied rate.

Agricultural practices and water management	Date	Day since sowing	Product	Commercial product rate	Active ingredient:	Applied rate
Pre-sowing fertilization	26 April	−15	Verdazoto		Dry manure: 12.5% N (11% organic N)	260 kg ha^−1^
Sowing	10 May	0				
Weed control pre-emergence	11 May	1	Ronstar	1 L/ha	Oxadiazon (380 g/L)	380 g/ha
Weed control post-emergence	8 June	30	Aura	0.6 L/ha	Profoxydim (200 g/L)	120 g/ha
			Facet	1.5 L/ha	Quinclorac (250 g/L)	375 g/ha
			Viper	1.5 L/ha	Penoxsulam (20 g/L)	30 g/ha
AWD irrigation and CF flooded	14 June	36				
AWD irrigation	20 June	42				
CF drained in preparation for fertilization	27 June	49				
Top-dress fertilization	30 June	52			(23−0−30)	300 kg ha^−1^
CF flooded and AWD irrigation	3 July	55				
Fungicide treatment	25 July	77	Amistar	1 L/ha	Azoxystrobin (250 g/L)	250 g/ha
AWD irrigation	31 July	83				
AWD flooded	8 August	92				
AWD and CF drained	26 August	110				
Harvest commenced	2 November	117				

### 2.2 Water management

Dry seeding took place on 10th May 2017 and both the AWD and CF plots were flooded to 5 cm above the soil surface on the 14th June; the AWD plots were allowed to naturally dry out while the CF plots were kept flooded. On a regular basis, the soil volumetric water content (VWC) at 10, 20, 30 and 40 cm (PR2 Profile Probe, Delta-T Ltd., Cambridge, UK), the water table depth (piezometer) and the soil matric water potential, at 25 cm depth, (Soilmoisture Equipment Corp. 30 cm) were monitored in every AWD plot (3 replicates in each AWD plot). The AWD cycles consisted of re-flooding the plots whenever the soil matric water potential reached −25 kPa (at 25 cm depth) and then allowed to dry out again.

### 2.3 Carbon dioxide measurements and environmental variables

Soil-atmosphere CO_2_ exchange was measured with an IRGA (EGM-4, PP-systems, Hitchin, UK) CO_2_ probe and temperature sensor fitted inside a clear, gas tight PVC cylindrical chamber (16 L volume and 196 L volume chamber used later in the season to accommodate the taller rice plants). The rate of CO_2_ accumulation was measured by placing the chambers over the rice plants for 3 min (5 min when using the larger chambers) with instantaneous CO_2_ concentrations (ppmv) measured every 5 s. No chamber bases were used due to the standing water in the rice paddy fields and in cases where there was no standing water present at the soil surface (during times of AWD), chambers were placed carefully on the soil surface and a skirt was applied to create an airtight seal. Net ecosystem exchange (NEE) was determined by using a clear chamber and ecosystem respiration (ER) measured by covering the chamber to create dark conditions. Gross primary productivity (GPP) was than calculated by subtracting NEE from ER. Measurements were taken weekly starting from day 70–119 since sowing.

Flux rates were determined using the *HMR* package (Pedersen et al., 2010) in R 3.0.2 (R Core Team, 2012) by plotting the best-fit lines to the data for headspace concentration (ppmv) against time (minutes) for individual fluxes. The Ideal Gas Law was then used to convert gas concentrations (ppmv) to moles of gas using the following equation:

(1) n=PV/RT

where n is the number of moles of CO_2_ gas (mol), P is atmospheric pressure (atm), V is the volume (L), R is the ideal gas constant (0.08205 L atm K^−1^ mol^−1^), and T is temperature (K). Fluxes were then reported in mg CO_2_-C m^−2^ h^−1^, and annual emissions were estimated by extrapolating each measurement to a 60 day period and summing for a year.

Soil temperature (at 10 cm and 20 cm depth) and soil moisture (at 10 cm depth) were simultaneously measured adjacent to the chambers using a ML2x ThetaProbe with 30 cm rods (Delta-T Ltd., UK) and type K thermocouples (Hanna Instruments Ltd., UK).

### 2.4 Soil CO_2_ partitioning

In order to create root free soil to determine heterotrophic respiration (RH), twenty-four (three per plot) soil cores lined with micro-pore mesh (50 × 50 μm) were inserted between rows of the rice plants. Soil cores (40 cm deep, 20 cm diameter) were removed and the mesh used to line the hole before placing the soil back to its original position, whilst keeping soil disturbance to a minimum. CO_2_ measurements were then taken on these root excluded collars at the same time as the ER CO_2_ measurements and autotrophic respiration (RA) was calculated by subtracting RH from ER.

### 2.5 Total, above and belowground biomass

Above (ANPP) and belowground biomass (BNPP) were estimated at key stages of plant growth for the individual cultivars, this included: tillering, panicle initiation, flowering and maturity. The season mean involved measuring each cultivar when it reached maturity. Belowground biomass was determined by collecting soil cores (15 cm depth by 10 cm width) using a root auger on all four of the chosen cultivars for the two treatments. Once collected, the soil was homogenized and roots were removed by hand over a 40-minute period, which was split into 10-minute intervals. Subsequently, the roots at each interval were cleaned of residual soil and detritus, dried at 70 °C and weighed. Saturation curves were fitted to the cumulative sampled dry root mass extracted against time for each core over a 12-hour period. The following equation was used to determine the saturation curve:

(2) $$R_{t}=R_{c}\;t/\left(k_{r}+t\right)$$

where R_t_ is the root mass extracted at time t; R_c_ is total root mass in the sample; k_r_ is the half saturation constant (Metcalfe et al., 2007).

Aboveground biomass was quantified by collecting the rice plants from directly where the soil corer was placed. The plants were dried at 65–70 °C for 48 h and weighed. When the grain started to develop at the later stages of plant growth, these were removed and weighed separately. NPP was estimated by using the total biomass (above and belowground) at the time of harvest.

### 2.6 Decomposition estimates

A decomposition experiment was set up as an additional estimate of soil organic matter mineralization, using Arelate rice straw. On 20th June 2017, 20 g of dry straw were, weighed and placed inside mesh bags (50 × 50 μm) and then buried at 10 cm depth in groups of 10, in each block (total 80 bags). A bag from each block was collected every week (8 bags), washed, dried and weighed to determine mass loss. The rate of decomposition was then calculated from the slope of a linear regression with time against mass loss.

### 2.7 Statistical analyses

Statistical analyses were performed using R version 3.0.2 (R_Core_Team, 2012). Extreme outliers (i.e. above 1000 ppm) were observed by visual inspection of the boxplots where points outside of the hinges (third quartile) were removed and the data were checked for normal distributions. To investigate the effects of treatment and cultivar on NEE, GPP and ER, a two-way ANOVA and Tukey's Honest Significant Different (HSD) post hoc test (*P* < 0.05) were initially conducted using water treatment and cultivar as independent variables and NEE, GPP and ER as the response variables, to examine statistically significant differences between means.

Further analysis using a mixed model restricted maximum likelihood analysis (REML) with repeated measures, was then computed using the *lme4* package (Bates et al., 2014), to identify any relationships between these response variables (NEE, GPP, ER) and extra environmental data measured (independent variables). The key independent variables included in the REML model included: water treatment, cultivar, growth stage, soil temperature (10 cm), soil volumetric water content, aboveground biomass and belowground biomass. This linear mixed model considered the violation of independence from repeating measurements on the same cores and also the nested design of the experiment (cultivar nested within the treatment plots). A Two-way ANOVA and Tukey's Honest Significant Difference (HSD) post hoc test (*P* < 0.05) was used to determine whether there was an effect of water treatment or stage of growth on the contribution of RA and RH to ER.

The effects of treatment, cultivar and growth stage on above and belowground biomass and yield were tested using a three-way ANOVA, which included treatment, cultivar, growth stage and their interaction as independent variables. Response variables included: total plant biomass (roots, straw, grain), aboveground biomass (straw + grain), belowground biomass (roots) and grain yield. Tukey's Honest Significant Difference (HSD) post hoc test (*P* < 0.05) was then used to determine any significant differences.

Simple linear regression analysis with time against mass loss was used to calculate the rate of decomposition of the litter bags and a two-way ANOVA, which included weight of leaf litter as the response variable and treatment and time as the independent variables, was used to determine any significant differences.

## 3 Results

### 3.1 NEE, GPP and ER

**Fig. 2:**
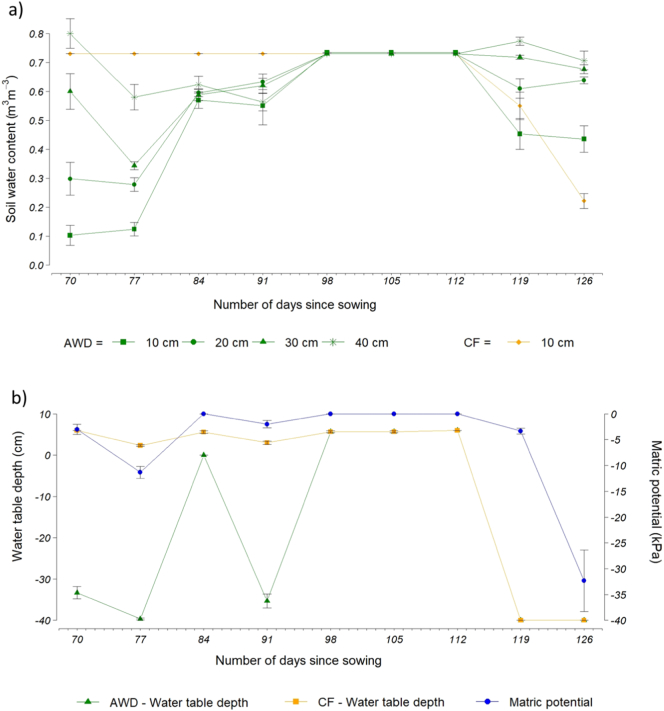
a. Top – mean soil water content (m3 m^−3^) on the AWD plots at 10, 20, 20 and 40 cm soil depth and on the CF plots at 10 cm soil depth for the period between July and September 2017. b. Bottom – mean water table depth (cm) on the left axis for the AWD and CF plots, and matric potential (kPa) on the right axis for the AWD plots. Error bars indicate standard 1 error of the mean.

In total there were 4 AWD cycles before keeping all the plots flooded at the commencement of flowering (8th–26th August), after which all the plots were dried in preparation for harvesting (2nd November) (Table 2 and Fig. 2a and b).

**Fig. 3:**
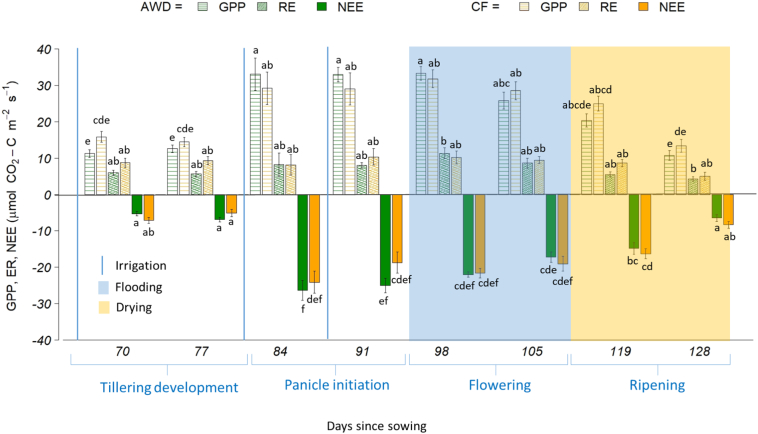
Gross primary productivity (GPP), ecosystem respiration (RE) and net ecosystem exchange (NEE) under alternate wetting and drying (AWD) and permanent flooding (CF) for the aggregated fluxes of the four cultivars during the season. Different lower-case letters represent significant differences (*P* < 0.05) among the four stages of growth and treatment for GPP, RE and NEE, separately. Errors bars indicate standard 1 error of the mean.

The results from the two-way ANOVA indicated that neither treatment nor cultivar had a significant effect on NEE and there was no interaction between the two variables. For the pooled data, NEE under AWD averaged −15.42 ± 0.96 μmol C m^−2^ s^−1^ (range: −37.0 to −0.67 μmol C m^−2^ s^−1^), while for CF, NEE averaged −14.66 ± 0.92 μmol C m^−2^ s^−1^ (range: −37.46 to −0.59) (Table 3). The results of the linear mixed effects model still indicated that neither treatment nor cultivar had a significant effect, but that plant growth stage and soil temperature significantly affected NEE with higher temperatures and larger plants causing more negative NEE (growth stage: F(19,3) = 33, *P*-value <0.001; soil temperature: F(19,1) = 18.4, *P*-value <0.001). The general trend for both the AWD and CF treatments was that NEE became more negative (i.e. increasing net C uptake) up to day 98, when the plants reached reproductive maturity and all the plots were flooded. This was followed by a gradual shift towards less negative values (i.e. decreasing net C uptake) up to day 119, when the plots were drained in preparation for harvesting (Fig. 3). NEE (C uptake) was the most negative during panicle initiation (days 81–95; −23.28 ± 1.91) and flowering (days 96–119; −20.00 ± 1.07), and the most positive during tillering (days 0–80; −6.13 ± 0.56) and ripening (119–135, −11.50 ± 1.21). Soil temperature affected NEE by increasing CO_2_ fluxes when the soils were warmer; i.e. the overall trend was towards more negative values (i.e. greater net C uptake) when temperatures were warmer. For instance, when temperatures reached their highest during flowering in August (~30 °C), NEE was also at its most negative. When comparing the soil temperature means between the AWD and CF treatments, no significant difference was observed (AWD = 24.9 ± 2.6, CF = 25.0 ± 25.1 °C).

**Table 3:** Mean net ecosystem exchange (NEE), gross primary productivity (GPP) and ecosystem respiration (ER) carbon dioxide (CO_2_) fluxes for the aggregated data set of the four cultivars at key phenological growth stages, and mean NEE for the four cultivars, individually. Different letters down the columns represent significant differences (*P* > 0.05) among the two treatments at different and growth stages. Standard errors indicate standard 1 error of the mean.

Stage of plant growth	Treatment	(μmol CO_2_–C m^−2^ s^−1^)			NEE (umol CO_2_–C m^−2^ s^−1^)			
		NEE	GPP	ER	Arelate	Gageron	Gleva	Prometeo
Tillering (0–80 days)	AWD CF	−6.12 ± 0.44^a^ −6.13 ± 0.67^a^	11.91 ± 0.72^a^ 15.14 ± 0.97^a^	5.79 ± 0.47^bc^ 9.01 ± 0.84^ab^	−4.25 ± 0.62^a^ −7.98 ± 1.38^ab^	−6.71 ± 0.84^a^ −6.84 ± 0.59^a^	−7.28 ± 0.61^a^ −5.73 ± 0.70^a^	−6.23 ± 1.04^a^ −4.00 ± 1.93^ab^
Panicle initiation (81–95 days)	AWD CF	−25.67 ± 1.64^ab^ −20.88 ± 2.17^b^	33.03 ± 2.31^b^ 29.11 ± 3.11^b^	8.06 ± 1.38^abc^ 9.68 ± 1.75^ab^	−27.33 ± 2.10^d^ −19.23 ± 6.21^bcd^	−24.96 ± 3.36^cd^ −26.09 ± 3.00^d^	−28.43 ± 2.80^d^ −18.56 ± 4.71^bcd^	−21.22 ± 4.99^c^ −19.65 ± 3.22^c^
Flowering (96–118 days)	AWD CF	−19.64 ± 0.93^c^ −20.35 ± 1.20^cd^	29.60 ± 1.65^b^ 30.16 ± 1.75^b^	9.95 ± 1.04^a^ 9.82 ± 0.95^ab^	−18.53 ± 2.48^bcd^ −24.56 ± 3.26^cd^	−22.29 ± 1.50^bcd^ −18.95 ± 0.89^bcd^	−21.42 ± 0.76^cd^ −21.92 ± 1.41^cd^	−16.33 ± 1.58^bc^ −15.95 ± 2.22^bc^
Ripening (119–135 days)	AWD CF	−10.68 ± 1.25^cd^ −12.32 ± 1.17^d^	15.54 ± 1.47^a^ 19.15 ± 1.80^a^	4.86 ± 0.49^c^ 6.83 ± 0.82^abc^	−12.16 ± 2.62^abc^ −12.58 ± 2.47^abc^	−13.54 ± 2.84^ab^ −16.00 ± 2.38^abc^	−9.33 ± 2.95^ab^ −12.24 ± 2.29^abc^	−7.67 ± 0.93^ab^ −8.47 ± 1.59^ab^
**Season mean**	AWD CF	−15.42 ± 0.96^a^ −14.66 ± 0.92^a^	22.41 ± 1.22^a^ 23.14 ± 1.17^a^	7.14 ± 0.48^a^ 8.77 ± 0.53^a^	−15.57 ± 2.02^a^ −15.95 ± 2.12^a^	−16.87 ± 1.87^a^ −16.57 ± 1.69^a^	−16.62 ± 2.06^a^ −14.44 ± 1.77^a^	−12.50 ± 1.71^a^ −11.68 ± 1.65^a^

When using a two-way ANOVA on the GPP data, the results also showed no significant effect of treatment, cultivar or their interaction on GPP. For the pooled data, GPP under AWD averaged 22.41 ± 1.22 μmol C m^−2^ s^−1^ (range: 54.41 to 3.87 μmol C m^−2^ s^−1^), while for CF, GPP averaged 23.14 ± 1.17 μmol C m^−2^ s^−1^ (range: 49.21 to 4.79) (Table 3). Analysis using a linear mixed effects model (with the same independent variables as the NEE analysis above) indicated that once again treatment and cultivar showed no significant effect but that growth stage and soil temperature significantly affected GPP (growth stage: F(16,1) = 6.0, *P*-value <0.01; soil temperature: F(16,1) = 14.0, *P*-value <0.001). The general trend for GPP followed that of NEE, with the most positive fluxes (net C uptake) during panicle initiation (days 81–95; 31.07 ± 2.71) and flowering (days 96–119; 29.88 ± 1.70), and least positive during tillering (days 0–80; 13.53 ± 0.85) and ripening (119–135, 17.35 ± 1.64) (Fig. 3). Higher GPP fluxes were also observed with higher soil temperatures.

Two-way ANOVA on the ER data also showed no significant effect of treatment, cultivar or interaction on ER. For the pooled data, ER under AWD averaged 7.14 ± 0.48 μmol C m^−2^ s^−1^ (range: 0.58 to 26.99 μmol C m^−2^ s^−1^), for CF, ER averaged 8.77 ± 0.53 μmol C m^−2^ s^−1^ (range: 0.54 to 30.34) (Table 3). The linear mixed effects model (again, using the same independent variables as NEE) indicated that only aboveground biomass (straw) significantly affected ER (F(16,4.5) = 1, *P*-value <0.03), with the biggest fluxes during panicle initiation (days 81–95; 8.87 ± 1.57 μmol C m^−2^ s^−1^) and flowering (days 96–119; 9.89 ± 1.00 μmol C m^−2^ s^−1^), when the plants were at their largest (Fig. 3).

### 3.2 RA, RH and decomposition rates

**Fig. 4:**
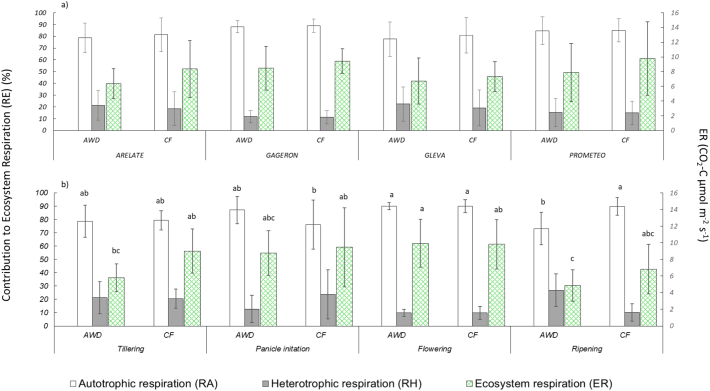
a. Top - contribution of heterotrophic respiration and autotrophic respiration to ecosystem respiration for the four cultivars. b. Bottom - contribution of heterotrophic respiration and autotrophic respiration to ecosystem respiration for the aggregated cultivar data set over the growing season. Different lower-case letters indicate significant differences (*P* < 0.05) among the different groups (a, cultivars and treatment, b: stages and growth and treatment) for heterotrophic respiration, autotrophic respiration and ecosystem respiration separately. Errors bars indicate standard 1 error of the mean.

RA was the dominant contributor to ER for all the cultivars under both treatments, accounting for 83 ± 8% of ER (data pooled between treatments and among cultivars; Fig. 4a). RA dominated ER throughout the growing season and at key stages of plant growth. In contrast, mean RH for the pooled data set was approximately 16 ± 8% of ER. At its highest, RH reached a maximum of only 29 ± 16% of ER in the CF plots, when the plants were tillering (i.e. day 77; Fig. 4b).

There was no significant difference in RA and RH between the CF and AWD treatments when the data were pooled across all plant growth stages (i.e. for RA, AWD = 82 ± 9% and CF = 84 ± 8%. For RH, AWD = 18 ± 9 and CF = 16 ± 8; Fig. 4a). However, we observed significant treatment effects during specific plant growth stages. For instance, during ripening, RA accounted for a significantly smaller proportion of ER in the AWD compared to the CF treatment (*P* < 0.05; AWD = 73 ± 12% versus CF = 90 ± 7%; Fig. 7b), whereas RH accounted for a significantly greater proportion of ER in the AWD compared to the CF treatment (*P* < 0.05; AWD = 27 ± 12% versus CF = 10 ± 7%). This significant difference between treatments was caused by a significant overall reduction in ER, and a shift in the relative proportions of RA and RH between the two treatments. In the AWD treatments, RA declined going from flowering to ripening, while RH showed the opposite trend (Fig. 4b). By contrast, in the CF treatments, neither ER, RA or RH showed a significant shift going from flowering to ripening. For example, ER in the AWD treatments declined from 9.89 ± 1.00 to 5.85 ± 0.66 μmol C m^−2^ s^−1^ going from flowering to ripening. Likewise, RA declined from 90 ± 3% during flowering to 73 ± 12% during ripening. In contrast, RH rose by 16%, going from 10 ± 3% during flowering to 27 ± 12% during ripening.

**Fig. 5:**
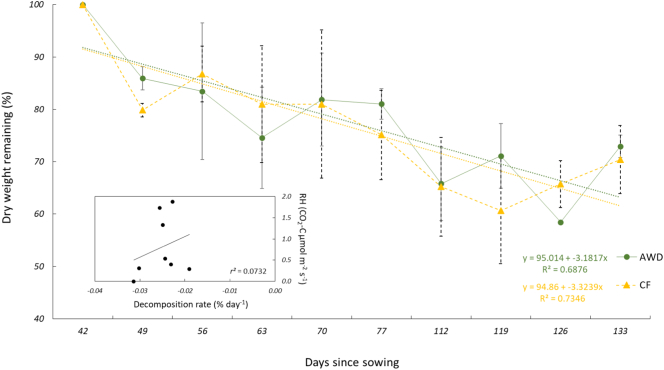
Mass losses (%) of rice leaf litter from the decomposition experiment (*n* = 4) on the alternate wetting and drying (AWD) and permanently flooded (CF) plots. Errors bars indicate standard 1 error of the mean and dotted lines show the regression slopes.

In the decomposition experiment, we found that approximately 42% of the buried rice straw in litter bags was lost over the 90-day incubation period (−0.03% decomposition rate day^−1^). There was no significant difference between the CF and AWD plots (AWD: y = 95.01–3.18×, CF: y = 94.86–3.32×) (Fig. 5), and decomposition was not a strong predictor of soil CO_2_ fluxes (RH) for the pooled dataset (*r*^*2*^ = 0.0732). Drainage did not appear to influence the decomposition rate-soil CO_2_ flux relationship.

### 3.3 Plant biomass, allocation and net primary productivity

**Fig. 6:**
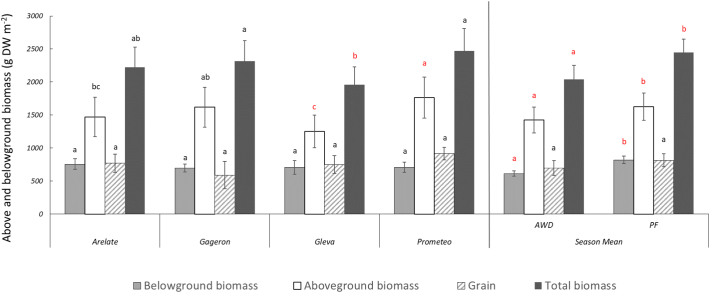
Mean aboveground (straw + grain) and belowground biomass (roots) for the four cultivars and the seasonal mean of the agregated data for the AWD and CF treatments. Different lower case letters represent significant differences (*P* > 0.05) among the cultivars and treatments for belowground, aboveground, total biomass, grain and seasonal mean, separately. Errors bars indicate standard 1 error of the mean.

**Fig. 7:**
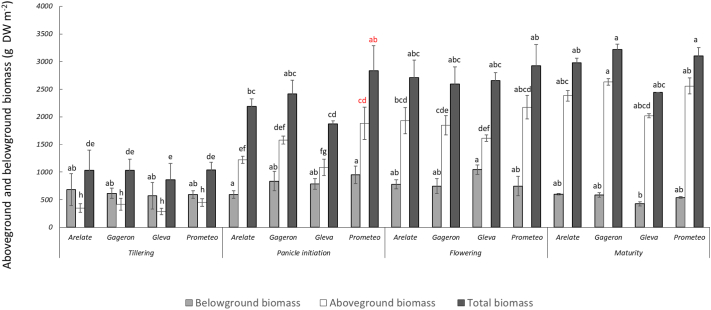
Mean aboveground (straw + grain) and belowground biomass (roots) for the four cultivars of the agregated data for the AWD and CF treatments at the four key growth stages. Different lower case letters represent significant differences (*P* > 0.05) among the different stages of growth, treatments and cultivars for belowground, aboveground and total biomass, separately. Errors bars indicate standard 1 error of the mean.

**Fig. 8:**
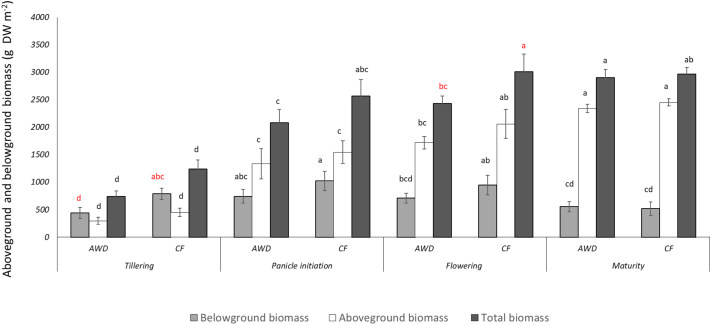
Mean aboveground (straw + grain) and belowground biomass (roots) for the two treatments using aggregated cultivar data at the four key growth stages. Different lower case letters represent significant differences (*P* > 0.05) among the different stages of growth and treatments for belowground, aboveground and total biomass, separately. Errors bars indicate standard 1 error of the mean.

NPP was significantly affected by cultivar (F(3,105) = 7.9, *p*-value <0.001), treatment (F(1,105) = 28.4, p-value <0.001) and stage of growth (F(3,105) = 128.7, p-value <0.001). For the effect of cultivar, we found that some plants showed significantly less total plant biomass than others (Fig. 6). For example, total plant biomass for Gleva (1957 ± 142 g m^−2^) was significantly lower than cultivars such as Gageron and Prometeo (2313 ± 167 and 2473 ± 173 g m^−2^, respectively). This difference was particularly observed during panicle initiation (Gleva: 1869 ± 200; Arelate: 2186 ± 201; Prometeo 2833 ± 323 g m^−2^) (Fig. 7). For the effect of treatment, we generally found that the CF treatment has slightly greater total biomass than AWD (CF = 2447 ± 113 g m^−2^ versus AWD = 2038 ± 112) (Fig. 6). This was specifically evident at the flowering growth stage where the total biomass was greater under the CF treatments (3009 ± 110) compared to the AWD treatments (2429 ± 57 g m^−2^) (Fig. 8). For the effect of growth stage, the overall trend was towards increasing total plant biomass gradually as the season progressed. Pair-wise comparisons (Tukey-Kramer HSD, *P* < 0.05) indicated significant differences among the growth stages, except during flowering and ripening, where differences were not statistically significant. Total plant biomass was lowest during tillering (991 ± 66 g m^−2^) and rose rapidly during panicle initiation (2325 ± 121 g m^−2^), reaching its largest during flowering (2719 ± 103 g m^−2^) and ripening (2934 ± 73 g m^−2^).

ANPP was significantly affected by cultivar and growth stage (cultivar: F(3,105) = 11.8, p-value <0.001; growth stage: F(3,105) = 181.7, p-value <0.001). For the effect of cultivar, we found that Gleva produced less aboveground biomass (AWD = 1247 ± 191; CF = 1253 ± 178 g m^−2^) compared to Gageron (AWD = 1545 ± 235; CF = 1692 ± 212 g m^−2^) and Prometeo (AWD = 1587 ± 213; CF = 1944 ± 236 g m^−2^). This was specifically observed during the panicle initiation growth stage (Prometeo: 1884 ± 240; Arelate: 1221 ± 126; Gleva: 1085 ± 199 g m^−2^) (Fig. 6, Fig. 7). For the effect of growth stage, the overall trend was an increase in aboveground biomass at every key growth stage; tillering had the smallest aboveground biomass (e.g. 376 ± 30 g m^−2^) and ripening the largest (2398 ± 52 g m^−2^). When analysing the grain separately to the straw, there were no statistically significant differences between cultivars or treatment.

BNPP was significantly affected by treatment, growth stage, and a treatment by growth stage interaction. For the effect of treatment (F(1,105) = 19.3, p-value <0.001), we found that belowground biomass was significantly lower in AWD (613 ± 31 g m^−2^) compared to CF (819 ± 43 g m^−2^) (Fig. 6). For the effect of growth stage ((F(3, 105) = 12.6, p-value <0.001), we found that root biomass tended to vary at different stages of plant growth. Belowground biomass was lowest during tillering (616 ± 76 g m^−2^), and highest during panicle initiation (883 ± 62 g m^−2^) and flowering (828 ± 66 g m^−2^). Belowground biomass was at intermediate levels during ripening (537 ± 28 g m^−2^). The three-way ANOVA indicated that root biomass was significantly different among all the different growth stages, except for panicle initiation and flowering which did not differ significantly from each other (Tukey-Kramer HSD, *P* < 0.05) (Fig. 7). We also found a weak but significant growth stage by treatment interaction ((F(3,105) = 3.3, p-value <0.02)); during tillering, AWD and CF treatments showed significant difference in belowground biomass (AWD = 443 ± 52 compared to CF = 788 ± 60 g m^−2^) (Fig. 8).

## 4 Discussion

### 4.1 No change in net ecosystem exchange and carbon storage with reduced water inputs

The rice paddies under both water management systems were net sinks of atmospheric C and did not differ significantly from each other in terms of NEE, GPP, RE or decomposition rates for the seasonal mean or during any of the key stages of plant growth measurement. Mean daily NEE in the CF rice paddy was −15.21 ± 0.95 g C m^−2^ d^−1^ (range:-38.87 to −0.61), and GPP 24.01 ± 1.20 g C m^−2^ d^−1^ (range: 51.07 to 4.97), affirming prior results of rice paddy studies using eddy covariance techniques in East Asia, India and the USA, where NEE estimates are between 5 and −39 and GPP between 5 and 50 g C m^−2^ day^−1^ (Alberto et al., 2009; Bhattacharyya et al., 2013; Miyata et al., 2005; Nay-Htoon et al., 2018; Saito et al., 2005; Swain et al., 2016). However, unlike in other studies where they reported a more positive NEE in intermittently flooded systems (Alberto et al., 2014; Liu et al., 2013; Miyata et al., 2000), mean daily NEE fluxes under AWD in this study were very similar to CF (−16.00 ± 1.00 g C m^−2^ d^−1^ (range: −38.39 to −0.70), and GPP 23.26 ± 1.27 g C m^−2^ d^−1^ (range: 56.46 to 4.02)), challenging our first hypothesis (**H1**). This is because ER was unchanged under more aerobic soil conditions, which also runs counter to what was expected in our second hypothesis (**H2**), but is supported by the results from decomposition experiment where no change was observed in decomposition rate##.

Rates of NEE and GPP were more affected by the specific stage of plant development and soil temperature, rather than with water management. There was a clear seasonal trend in CO_2_ fluxes, with more negative NEE values (i.e. increasing net C uptake) observed as the rice plants reached heading to flowering growth stage, followed by a steep decline in net C uptake (i.e. less negative NEE values) as the plants reached maturity. These results are consistent with other rice studies, and are explained by an increase in GPP as aboveground plant biomass and leaf area index (LAI) increases as plants reach heading and flowering growth stages (Alberto et al., 2009; Campbell et al., 2001; Miyata et al., 2005; Saito et al., 2005). This is subsequently followed by a decline in GPP towards the ripening growth stage due to leaf senescence or reduction in leaf greenness (Pakoktom et al., 2009; Swain et al., 2016). Factors such as temperature and light play an important role in regulating rates of ER, NEE and GPP, with peaks in temperature and light availability during July and August facilitating high rates of C uptake during heading and flowering. (Krishnan et al., 2011; Wohlfahrt and Gu, 2015; Xin et al., 2017). One potential issue is that the relationships between growth stage or temperature and C fluxes could be confounded, because mean air temperatures are generally warmer during later phases of plant growth.

Unlike NEE and GPP, ER was more affected to changes in aboveground biomass, but not to plant growth stage or temperature. Autotrophic respiration dominated ER in this study site (AWD = 82% and CF = 84% of ER), with RH accounting for a much smaller proportion (AWD = 18% and CF = 16% of ER) of ER. The proportion of RH from our CO_2_ partitioning experiment was within the range (16 ± 8% of ER) of other cropland systems (5–40%), so these data are not unusual in and of themselves (Hanson et al., 2000; Suleau et al., 2011; Swinnen, 1994). Our findings are also broadly in agreement with other paddy studies that have sought to partition RA and RH by measuring soil CO_2_ flux between unvegetated plant rows (i.e. RH ranging from 0.02 to 3.91 g C m^−2^ d^−1^) (Iqbal et al., 2009; Liu et al., 2013; Nishimura Seiichi et al., 2014), emphasizing the important role of plant metabolism in modulating ER and NEE in rice systems. While it is somewhat surprising that temperature did not play a more important role in regulating ER, the relative importance of aboveground biomass and RA in determining rates of ER suggests that ER during the growing season may be more strongly determined by the growth and activity of the plant community, rather than by abiotic variables such as temperature.

Results for our AWD treatment are novel and important from a climate change mitigation perspective, because these data imply that soil and ecosystem C stocks in European rice soils are less likely to be destabilised by a shift towards less water-intensive production systems, such as AWD or other forms of intermittent drainage. Thus, in addition to the benefits of reduced emissions of CH_4_ under AWD, there is no additional risk of enhanced soil C loss, which could offset the potential climate benefits of AWD in a European context. However, our results are also surprising because other intermittent drainage studies suggest that aerobic soil conditions can enhance ER (Liu et al., 2013; Nishimura Seiichi et al., 2014), with some paddy systems changing from a net sink to a source of CO_2_ with increased soil drainage (Miyata et al., 2000)_._ Our findings also run counter to expectations from other human-affected temperate wetlands (e.g. managed peatlands), where investigators have observed enhanced SOM mineralization and ecosystem C loss following drainage (Boyd, 1995; Hooijer et al., 2010; Jungkunst Hermann and Sabine, 2007; Moore and Dalva, 1993). One possible explanation is that the 1–2 week wetting-drying cycle for our AWD system was not sufficient to cause an observable shift in the carbon metabolism of the soil; this interpretation is supported by findings from our leaf litter decomposition experiment, which showed no significant difference in decay rates between AWD or CF treatments, implying that the underlying carbon metabolism of the soil was not altered by AWD. Alternatively, it is possible that the low C stock of these soils (1.3%, 13.66 ± 3.32 g Kg^−1^) meant that there was relatively little labile OM to oxidise or that the quality (i.e. relative lability of the SOM) was too poor to support high rates of RH, even under more aerobic conditions (Muhr et al., 2011; Swails et al., 2019). However, this interpretation of the data is not fully supported by the results of the decomposition experiment; under a situation where soil RH is constrained by a combination of both low redox potential and labile C availability, then one would predict that alleviation of both these conditions would lead to a significant increase in rates of organic matter utilization. Yet in the decomposition experiment, we did not see a significant increase in decay rates of rice straw under AWD.

### 4.2 Effects of water management and cultivar on plant productivity, allocation and yield

Even though NEE, GPP, ER and decomposition rates did not differ significantly among treatments or cultivars, we did observe differences in plant productivity and allocation. Crucially, however, yield was not significantly impacted by water management, partially supporting **H3**. The overall trend was towards slightly greater total plant biomass in the CF compared to AWD treatments, partially falsifying **H3**. No significant effect of AWD on yield has also been observed in an experiment in Bangladesh. However, they also recorded consistently higher harvest index values on plants grown under AWD, which was attributed to a change in the allocation of resources, with either the number of tillers or productive tillers increasing with drier conditions (Norton et al., 2017a, Norton et al., 2017b). Differences between water treatments were particularly evident during the flowering stage of growth, when differences in total plant biomass were most pronounced between water treatments.

Interestingly, the differences in total plant biomass and productivity were attributable to differences in belowground biomass and productivity (BNPP) between water treatments, rather than due to differences in aboveground biomass and productivity (ANPP), challenging **H4**. Contrary to expectation, ANPP did not differ significantly among water treatments, whereas BNPP was significantly lower in AWD compared to CF treatments. We predicted that water stress might inhibit leaf production and cause a decline in leaf area, leading to retarded leaf growth and light interception, and hence reduce ANPP (Lilley and Fukai, 1994). Likewise, in-line with plant allocation theory, we predicted that the plants would allocate more energy and resources to roots over shoots, in response to reduced water supply (Sandhu et al., 2017; Zhang et al., 2009). Contrary to expectation, we observed the opposite of these trends. We suspect that root growth may have been restricted because AWD facilitated particle cementation and soil compaction in the silty clay loam soils found at this study site, inhibiting root growth (Rao and Revanasiddappa, 2006). In 2015, a penetrometer survey was carried out on the same site as this study to determine whether the load-bearing capacity of the soil was affected by periods of draught. The results showed that the top 15–30 cm were harder under AWD (1254 ± 167 KPa) compared to CF (807 ± 111 kPa) and that once the plots were reflooded, the soils did not recover back to their original softness. However, the soils were not hard enough to be considered impactful on root growth. Alternatively, it is possible that roots in the AWD treatment may have been growing more laterally or vertically (i.e. >15 cm; below the sampling depth utilised in this study) (Gu et al., 2017), and were not representatively sampled by our sampling methodology.

In terms of the effect of cultivar, all of the cultivars showed similar trends in productivity, allocation and yield, except the Gleva cultivar. Gleva consistently showed lower aboveground biomass and ANPP at all growth stages compared to the other cultivars. For all four cultivars on both treatments, there was a rapid increase in ANPP as the rice reached flowering stage, correlating with GPP. The only significant difference observed was a lower ANPP in Gleva at all growth stages, compared to the other cultivars. However, even though GPP is primarily controlled by LAI, the significantly lower aboveground biomass in Gleva was not enough to significantly reduce GPP. On average among the four cultivars, AWD delayed maturity by only two days; previous fields trials have shown delays up 17 days, but similar to this study, no significant loss in yields were reported (Howell et al., 2015; Sudhir-Yadav Gill et al., 2011). The BNPP followed a different trend to ANPP, where BNPP on all the cultivars increased up to panicle initiation and then declined during flowering and even further at ripening. In other rice studies, the proportion of photosynthetic C allocated underground has also been documented to significantly decrease after tillering to maturity (Watanabe et al., 2004).

## 5 Conclusions

This study demonstrates that using water saving techniques such as AWD could be a workable solution for sustainable and environmentally friendly rice cultivation in Northern Italy and potentially in the rest of southern Europe, without the associated risks of enhancing C losses from aerobic SOM decomposition or compromising crop yield. By determining the effects of AWD on ecosystem C dynamics, we were able to establish the underlying mechanistic basis as to why no C losses were observed. We hypothesised that NEE would be more positive under AWD compared to CF due to increased ER (in particular RH) under aerobic soil conditions (**H1 and H2**). However, these hypotheses were rejected because NEE, ER and RH were significantly unaffected by AWD conditions. We also hypothesised that NPP and grain yield would be similar in AWD compared CF (**H3**). This was confirmed with yield showing no effect but greater plant biomass (NPP) was observed under AWD. Interestingly BNPP was reduced while ANPP increased in AWD compared to CF, challenging plant resource allocation theory (**H4**). In our system, the main driving factors for C dynamics were ANPP and soil temperature and not RH as previously thought. Whilst the overall investment of C to the root system was reduced under AWD for the four rice cultivars, there was no effect of treatment on the aboveground biomass or yield, suggesting only partial stress on the rice plants under these controlled levels of water reduction. Our study therefore highlights the importance of using ‘safe’ AWD and calls for further research to push these boundaries and assess the impact of longer cycles of AWD on C dynamics and over multiple years, considering our growing global need to conserve water. Additional studies are also needed to incorporate a range of organic rich paddy soils to determine whether soil C metabolism will increase when more labile organic carbon is available.

## Declaration of Competing Interest

None.
